# Different Growth and Physiological Responses of Six Subtropical Tree Species to Warming

**DOI:** 10.3389/fpls.2017.01511

**Published:** 2017-08-29

**Authors:** Yiyong Li, Guoyi Zhou, Juxiu Liu

**Affiliations:** ^1^School of Forestry and Landscape Architecture, Anhui Agricultural University Hefei, China; ^2^Key Laboratory of Vegetation Restoration and Management of Degraded Ecosystems, South China Botanical Garden, Chinese Academy of Sciences Guangzhou, China

**Keywords:** climate warming, tree growth, subtropical forest, stomatal traits, non-structural carbohydrates

## Abstract

Quantifying changes in interspecific plant growth and physiology under climate warming will facilitate explanation of the shifts in community structure in subtropical forest. We evaluated the effects of 3 years climate warming (ca. 1°C, 2012–2015) on plant growth and physiological parameters of six subtropical tree species by translocating seedlings and soil from a higher to a lower elevation site. We found that an increase in soil/air temperature had divergent effects on six co-occurring species. Warming increased the biomass of *Schima superba* and *Pinus massoniana*, whereas it decreased their specific leaf area and intrinsic water use efficiency compared to other species. Warming decreased the foliar non-structural carbohydrates for all species. Our findings demonstrated that a warmer climate would have species-specific effects on the physiology and growth of subtropical trees, which may cause changes in the competitive balance and composition of these forests.

## Introduction

Tropical and subtropical forest ecosystems occupy large areas of the global surface and provide important ecosystem services, such as carbon storage, global biogeochemical cycles and the conservation of biodiversity. The impacts of climate warming (Pachauri et al., 2014), however, are gradually representing a prominent disturbance, affecting from individuals to whole ecosystems (Arend et al., 2011). Numerous studies of subtropical and tropical forests have reported that climate warming is changing forest structure and tree mortality (Zhou et al., 2013, 2014). Shifts in plant community composition, plant cover and growth (increases in plant biomass) will affect the vegetation feedbacks to climate change.

Many temperature manipulations have been conducted to quantify growth and physiological responses (Gimeno et al., 2012; Huang et al., 2015). However, because most studies focus on responses of individual tree species, we have little understanding of how warming would affect co-occurring species. Competition with neighboring plants is very common in various ecosystems (Wang et al., 2012; Wang Y. et al., 2016), so that the competitive hierarchies of co-occurring species may re-ranked by divergent morphological and physiological responses under warming (Hikosaka et al., 2005).

Many warming experiments have been conducted in temperate ecosystems where plant growth may be temperature-limited (Way and Oren, 2010). Increased biomass production of tree seedlings under elevated temperature were observed in these warming experiments (Ghannoum et al., 2010; Way and Oren, 2010; Sendall et al., 2014), although responses were often small or transient and varied across species and study site (Lin et al., 2010). However, the responses of tropical and subtropical tree species to climate warming remain unexplored. Several studies suggested that tropical tree species which have narrower temperature tolerance may be more susceptible than temperate tree species under future warming scenarios (Sala et al., 2000; Cunningham and Read, 2002). In addition, biomass allocation patterns may be altered when plants are exposed to warming (Kasurinen et al., 2016; Wang P. et al., 2016). However, there are quite few experiments which have been carried out in tropical and subtropical areas, and responses of plant growth in tropical and subtropical forest ecosystems remain poorly resolved.

Plant growth may be affected directly by response of biochemical (i.e., photosynthesis) (Smith and Dukes, 2013) and physiological processes (i.e., stomatal conductance) (Zheng et al., 2013), or indirectly by changes in nutrient and water availability (Jónsdóttir et al., 2005). In connection with plant growth and carbon assimilation, stomatal traits including the length and density of stomata, which can determine the maximum stomatal conductance to CO_2_ and H_2_O (Franks and Beerling, 2009), could change to optimize their gas exchange (Zhang et al., 2010). Changes in stomatal apertures and density are often associated with changes in water-use efficiency (Franks et al., 2015) which can affect plant growth and water stress (Han et al., 2013). It is widely recognized that plants must achieve a balance between carbon assimilation, carbon storage and growth, all of which are directly or indirectly affected by climate warming (Smith and Stitt, 2007). The concentration of non-structural carbohydrates (NSC) within plant tissues, which depends on the balance between carbon supply (i.e., photosynthesis) and carbon demand (i.e., growth) (Michelot et al., 2012), were considered to decrease under short-term warming (Huang et al., 2015). Therefore, it is necessary to study the interaction among carbon assimilation rate, plant biomass and NSC under warming.

The coniferous and broad leaved mixed forest represents one of the most widespread secondary vegetations and developed well in southern subtropical China. Shifts in the structure and distribution of these forests under climate warming are likely to have important consequences. Here, we conducted a downward translocation (translocate soil and trees from 300to 30 m a.s.l) experiment to examine the effects of elevated temperature on tree growth and physiological performance of six subtropical tree species in a mixed forest. In light of previous studies (Li et al., 2016a,b), we tested the following hypotheses: (1) the six species would show divergent responses of tree growth and biomass allocation pattern, and (2) warming cause adjustments in plant morphology and physiology to support growth.

## Materials and Methods

### Study Site

We conducted this research at Dinghushan Biosphere Reserve, an UNESCO / MAB site located in the central Guangdong Province in southern China (112°10′E, 23°10′N). The local climate is typical south subtropical monsoon, with mean annual precipitation of 1956 mm and mean annual temperature of 21°C. The bedrock is sandstone and shale. Soils are classified as ultisols with a pH 4.0–4.9 in the top 5 cm.

### Translocation Experiment

We selected two field translocation sites, one control site and one warm site (at 300 m and 30 m a.s.l, respectively). At each site, we selected three 3 × 3 m plots in an open area and then shielded below-ground (0.8 m deep) with concrete brick wall bonding with ceramic tile, leaving one hole connected with PVC tube at the bottom and the top of the wall to collect underground water and surface runoff, respectively. In April 2012, soil and 1-year-old seedlings were collected from a coniferous and broadleaved mixed forest that is near the control site. Three different layers of soils (0–20, 20–40, and 40–70 cm) were homogenized separately. Seedlings were stored in shade containers with soil from the collection sites. In May 2012, three different layers of soils were transferred into the plots correspondingly. The seedlings were transplanted into the plots in a randomized block design (*n* = 6 replicates per species).

The six species included in this study were specifically selected due to their common occurrence and distribution range (existence in almost all regions along the altitudinal gradient) from the mixed forest. They included *Schima superba* Gardn. et Champ, *Syzygium rehderianum* Merr. et Perry, *Machilus breviflora* (Benth.) Hemsl, *Pinus massoniana* Lamb., *Castanopsis hystrix* Hook. f. et Thomson ex A. DC, *Ardisia punctata* Lindl. All species were evergreen, ensuring that their leaves are exposed to the full seasonal changes of temperature. Coniferous and broadleaved species (*P. massoniana* vs other species) were chosen.

Each plot was installed with a meteorological station to record air temperature (HMP155A, Vaisala, Finland). We continuously monitored soil temperatures and soil moisture (0–10 cm depth) using automated sensors (CT 109, CS616 and CR1000 Data Loggers, Campbell, United States). More detailed information about the collection and establishment of the experiment has been reported previously (Li et al., 2016a,b).

### Growth Measurements and Sample Collection

Tree height and basal diameter were measured at the time of planting in May 2012 and then assessed in June annually. Plant height was measured as the distance between the soil surface and the tip of the apical bud. The basal diameter was assessed at the soil surface. One tree per species in each chamber was destructively harvested in June 2014 and June 2015. The soil on the roots was carefully removed, and any root material in the soil was also collected. The fresh weights of all leaves, stems and branches, and roots of the harvested trees were measured. Then samples of leaves, stem and branches, and roots were collected to calculate dry/fresh ratio (oven-dried at 65°C until constant weight / fresh weight). The biomass of each harvested tree was calculated with fresh weights of all organs and dry/fresh ratio. Strong correlations among dry biomass for each component part, basal area and height existed irrespective of size in trees harvested, which were stated in the equation:

$$B=a\times (D^{2}H)+b$$

Where *B* is dry biomass of each tree components including root, stem and leaf (g m^-2^), *a*, *b* represent the regression coefficients, *D* is plant basal diameter (cm), *H* is tree height (cm), and *a, b* are regression coefficients (See Supplementary Table S1). The biomass of other un-harvested tree was calculated separately using their height and basal diameter and the allometric relationship described by the equation above.

In June 2015, total plant leaf area was determined by a portable leaf area meter (LI-3100A, Li-Cor, United States). For each harvested seedling, specific leaf area [Specific leaf area (SLA); leaf area/leaf biomass. cm^2^g^-1^] and leaf area ratio [leaf area ratio (LAR); total leaf area/total plant biomass, cm^2^g^-1^] were calculated. As *P. massoniana* is a needle-leaved species which could not be measured for leaf area, we only studied the other five species for SLA and LAR in our experiment. The whole plant biomass, the fraction of biomass allocated to roots [root mass ratio (RMR); roots mass fraction], leaves [leaf mass ratio (LMR); leaves mass fraction] and stems [shoot mass ratio (SMR); stems mass fraction] and the roots to shoots ratio (R/S; roots biomass/shoots biomass) were also calculated.

### Leaf C Isotope Discrimination and Non-structural Carbohydrate (Soluble Sugar and Starch)

Leaf samples from the destructive harvests in June 2015 were oven dried at 70°C and ground to powder to analyze leaf C isotope discrimination (δ^13^*C*) and soluble carbohydrates. The leaf C isotope discrimination (δ^13^*C*) has been increasingly accepted as an index to infer intrinsic water use efficiency (Dawson et al., 2002). The C stable isotope composition was obtained by mass spectrometry (Finnigan Mat, Delta S, Bremen, Germany) in the public laboratory of Southern China Botanical Garden, Chinese Academy of Sciences, with Pee Dee Belemnite as standard. The δ^13^*C* (in parts per thousand, ‰) was as (Farquhar et al., 1989):

$$\delta^{13}C=(R_{\mathrm{sample}}/R_{\mathrm{standard}}-1)\times 1000$$

where *R*_sample_ and *R*_standard_ are the ^13^*C*:^12^*C* ratios of the leaf sample and the ^13^*C*/^12^*C* ratio of the international Pee Dee Beleminite (PDB) standard.

Methods of soluble sugar and starch assay were described in Mitchell et al. (2013) with some modifications. Soluble carbohydrates were extracted using an ethanol technique and determined using the anthrone colorimetric assay (Ebell, 1969). The concentrations were calculated by comparing with glucose standards, expressed as mg glucose g^-1^DW.

### Stomatal Length and Stomatal Density

In June 2014 and 2015, three leaves per tree were collected from all tree species except for *P. massoniana* in each chamber. Leaf epidermises on the adaxial side were taken centrally in the leaf midway. The epidermises were mounted on a microscope slide and observed using a light microscope (DM2500, Leica, Germany). For each epidermal peel, 20 stomata were sampled to measure length and 3 fields (300 μm × 300 μm) were sampled for density. As stomatal traits of *P. massoniana* could not be measured using this method, we only studied other five species for stomatal traits.

### Statistical Analysis

Basal diameter, tree height as well as stomatal traits were analyzed using a repeated-measures analysis of variance (RM-ANOVA) with warming (referred to downward translocation), species and their interaction as independent factors. Two-way ANOVA was used to assess the effects of warming, species and their interaction on other growth and physiological traits. Data analyses were carried out using SPSS 17.0 (SPSS Inc., Chicago, IL, United States). *T*-test was also used to analyze the significant differences in these parameters as well as soil moisture and temperature between warm and control site. All analyses were conducted using SPSS 17.0 (SPSS Inc., Chicago, IL, United States). Variables normality and residual homocedasticity were checked.

## Results

### Soil Temperature and Soil Volumetric Water Content

**FIGURE 1:**
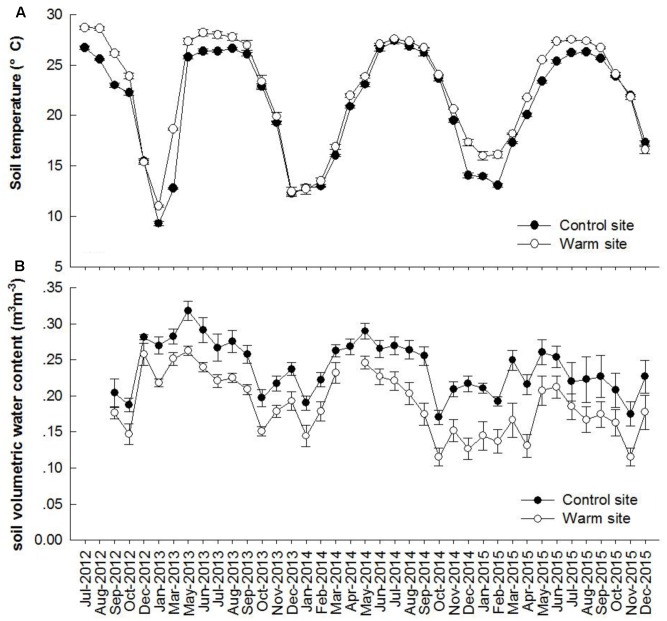
Dynamics of soil temperature **(A)**, volumetric water content **(B)** at 10 cm depth in the control and warm sites from July 2012 to December 2015. Error bars are standard error.

Soil temperature and soil moisture exhibited clear seasonal patterns (**Figure 1**). From July 2012 to December 2015, monthly soil temperature was on average 1.27°C higher under warming (*p* < 0.05, **Figure 1A**). From September 2012 to December 2015, mean soil volumetric water content were 0.19 and 0.23 m^3^m^-3^ in the warm and control sites, respectively (*p* < 0.05, **Figure 1B**).

### Tree Growth and Biomass Allocation

**FIGURE 2:**
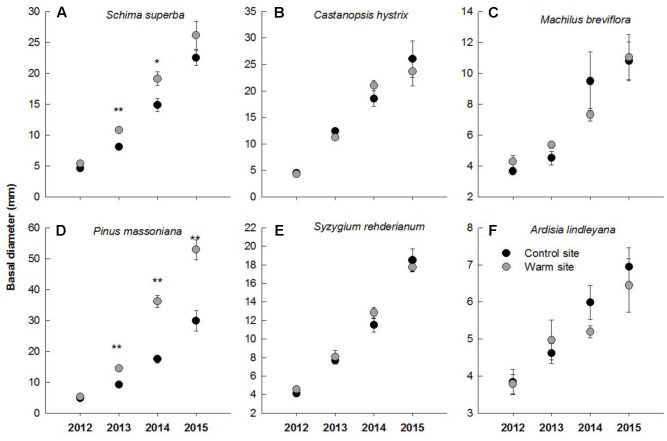
Basal diameter of six tree species in the control and warm sites from June 2012 to June 2015. *Schima superba* **(A)**, *Castanopsis hystrix* **(B)**, *Machilus breviflora* **(C)**, *Pinus massoniana* **(D)**, *Syzygium rehderianum* **(E)**, and *Ardisia lindleyana* **(F)**. Error bars are standard error. ^∗^*p* < 0.05; ^∗∗^*p* < 0.01.

**FIGURE 3:**
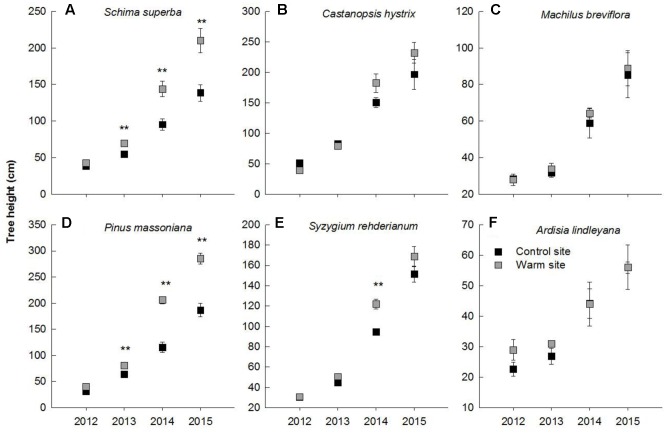
Tree height of six tree species in the control and warm sites from June 2012 to June 2015. *Schima superba* **(A)**, *Castanopsis hystrix* **(B)**, *Machilus breviflora* **(C)**, *Pinus massoniana* **(D)**, *Syzygium rehderianum* **(E)**, and *Ardisia lindleyana* **(F)**. Error bars are standard error. ^∗^*p* < 0.05; ^∗∗^*p* < 0.01.

**FIGURE 4:**
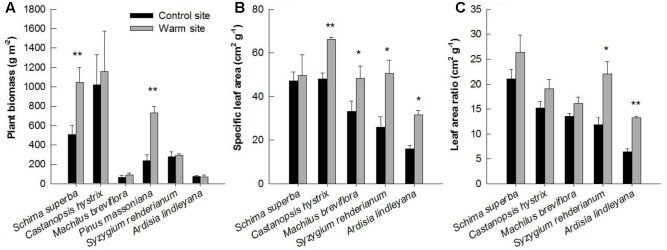
Plant biomass **(A)**, specific leaf area **(B),** and leaf area ratio **(C)** of six species in the control and warm sites in June 2015. Error bars are standard error. ^∗^*p* < 0.05; ^∗∗^*p* < 0.01.

In general, warming, species and their interactions had significant effects on basal diameter, height and biomass over the entire experimental period (**Table 1**). Warming significantly increased basal diameter of *S. superba* (+33 and +28% in 2013 and 2014, respectively) and *P. massoniana* (+57, +106, and +77% in 2013, 2014, and 2015, respectively) (*p* < 0.05, **Figures 2A,D**) (**Figure 2**); however, it had no significant effect on other tree species. Tree height of *S. superba* (+28, +51, and +51% in 2013, 2014, and 2015, respectively), *P. massoniana* (+27, +78, and +53% in 2013, 2014, and 2015, respectively) and *S. rehderianum* (+29% in 2014) were significantly higher under warming (*p* < 0.05, **Figures 3A,D,F**) (**Figure 3**). In 2015, warming significantly increased whole plant biomass of *S. superba* and *P. massoniana* by 107% and 206%, respectively (*p* < 0.05), but did not significantly affect other tree species (*p* > 0.05) (**Figure 4A**).

**Table 1:** Effects of warming, species and their interactions on growth and physiological parameters.

Variables	Warming	Df	Species	Df	Warming^∗^Species	Df
Stem diameter	25.0***	1	101.6***	5	17.4***	5
Tree height	49.6***	1	101.2***	5	7.9***	5
Stomatal length	16.8**	1	173.8***	4	0.6	4
Stomatal density	6.4*	1	28.8***	4	0.9	4
Biomass	6.4*	1	15.1***	5	3.0*	5
Root mass ratio	0.1	1	398.9***	5	5.4**	5
Stem mass ratio	1.4	1	545.1***	5	0.5	5
Leaf mass ratio	0.2	1	58.4***	5	4.5**	5
Root / Shoot	0.5	1	668.9***	5	2.7*	5
Specific leaf area	83.9***	1	44.5***	4	4.7**	4
Leaf area ratio	26.1***	1	15.7***	4	1.4	4
Leaf δ^13^*C*	4.7*	1	69.2***	5	8.0***	5
Soluble sugar	23.9***	1	15.7***	5	0.9	5
Starch	21.8***	1	93.6***	5	8.1***	5


*Numbers and dfs are *F*-values and their degrees of freedom, respectively. Asterisks indicate the level of significance (no asterisk = not significant, ^∗^*p* < 0.05, ^∗∗^*p* < 0.01, ^∗∗∗^*p* < 0.001).*

**FIGURE 5:**
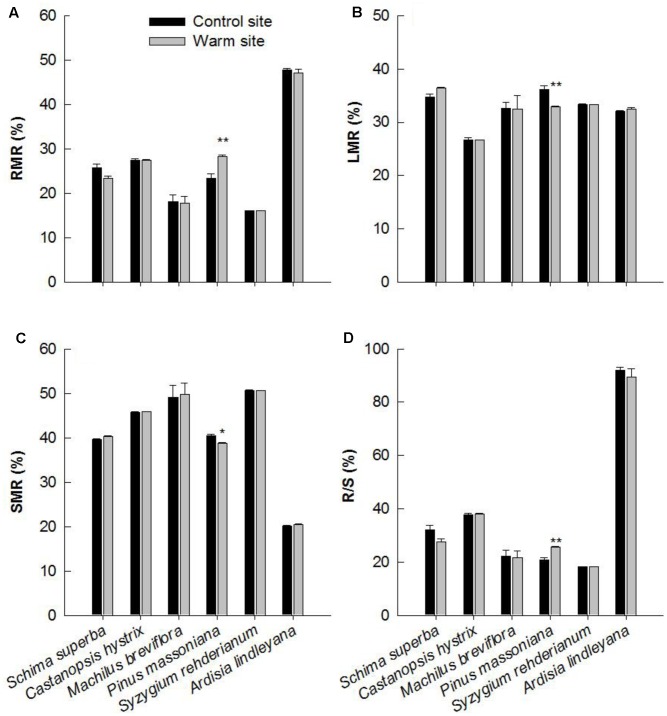
Plant biomass allocation of six species at warm and control site in June 2015. **(A)** RMR: root mass ratio, **(B)** LMR: leaf mass ratio, **(C)** SMR: stem mass ratio, **(D)** R/S: root/shoot mass ratio. Error bars are standard error. ^∗^*p* < 0.05; ^∗∗^*p* < 0.01.

Except for *P. massoniana*, warming had no significant effect on biomass allocation patterns in June 2015 (**Table 1**). *P. massoniana* had greater RMR and root/shoot (R/S), lower LMR and SMR under warming (*p* < 0.05, **Figure 5**).

Specific leaf area and LAR were significantly affected by downward translocation in June 2015 (**Table 1**). Except for *S. superb*, SLA of other species was significantly higher in the warm sites compared to those in the control site for all species (*p* < 0.05) (**Figure 4B**). LAR of *S. rehderianum* and *A. lindleyana* in the warm site were significantly greater than that in the control site (*p* < 0.05, **Figure 4C**).

### Leaf δ^13^*C*

**FIGURE 6:**
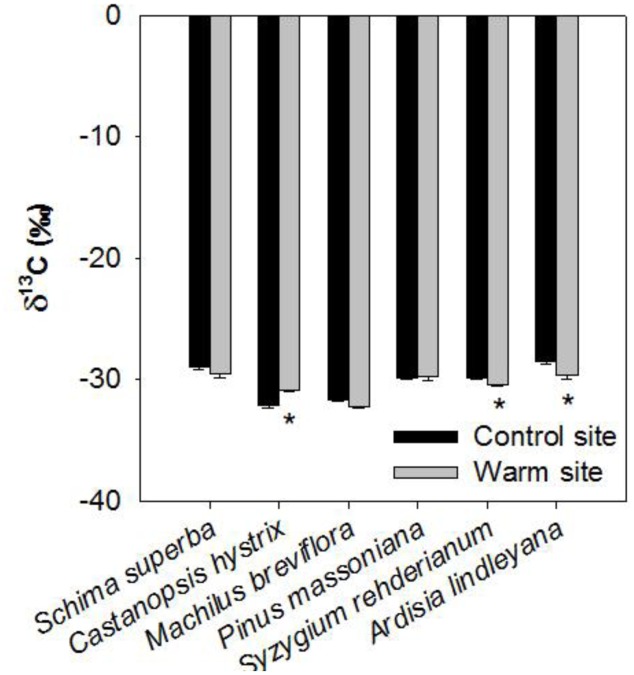
Carbon isotope composition in leaves of six tree species in the control and warm sites in June 2015, respectively. Error bars are standard error. ^∗^*p* < 0.05; ^∗∗^*p* < 0.01.

Warming, species and their interactions significantly affected leaf δ^13^*C* in June 2015 (**Table 1**). Leaf δ^13^*C* of *S. rehderianum* and *A. lindleyana* was significantly lower under warming, but leaf δ^13^*C* of *C. hystrix* was significantly higher under warming (*p* < 0.05, **Figure 6**).

### Non-structural Carbohydrates

**FIGURE 7:**
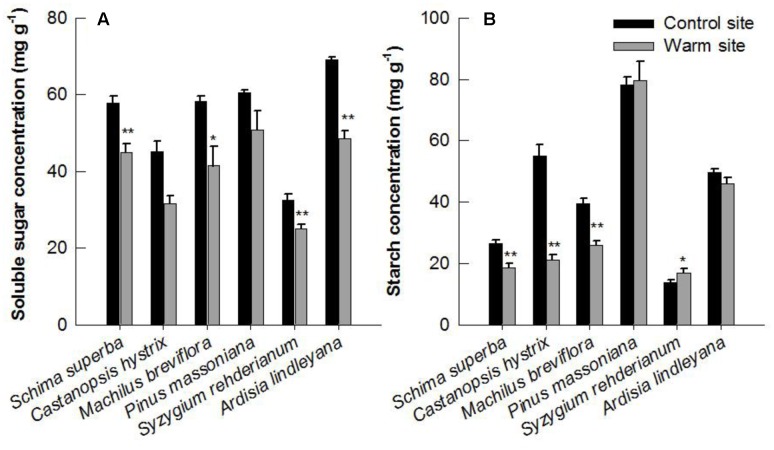
Soluble sugar **(A)** and starch concentration **(B)** in leaves of six species in the control and warm sites in June 2015. Error bars are standard error. ^∗^*p* < 0.05; ^∗∗^*p* < 0.01.

Leaf soluble sugar and starch varied significantly between species and were all affected significantly by warming in June 2015 (**Figures 7A,B** and **Table 1**). The soluble sugar of *S. superba*, *S. rehderianum*, *C. hystrix* and *M. breviflora* was 22, 23, 30, and 29% lower under warming, respectively (*p* < 0.05, **Figure 7A**). The starch of *S. superba*, *C. hystrix*, and *M. breviflora* was 30, 62, and 35% lower under warming, respectively (*p* < 0.05, **Figure 7B**).

### Stomatal Length and Stomatal Density

Stomatal length and stomatal density varied significantly between tree species and were affected significantly by warming (**Tables 1**, **2**). Warming significantly decreased stomatal density of *S. superba* and *C. hystrix* by 17 and 30% in 2015 (*p* < 0.05).

**Table 2:** Stomatal length and stomatal density in five tree species in warm and control site in June 2014 and 2015.

Year	Species	Site	Stomatal length (μm)	Stomatal density (stomata/mm^-2^)
2014	*Schima superba*	Control	12.11 ± 1.67	438.8 ± 119.2
		Warm	11.90 ± 1.28	442.5 ± 82.7
2015		Control	13.56 ± 0.91	509.8 ± 67.9^∗^
		Warm	12.37 ± 0.70	424.6 ± 15.4
2014	*Syzygium rehderianum*	Control	12.57 ± 3.24	348.1 ± 44.1
		Warm	10.74 ± 1.44	345.6 ± 63.6
2015		Control	10.53 ± 0.09	412.3 ± 50.2
		Warm	10.59 ± 0.42	440.4 ± 49.7
2014	*Castanopsis hystrix*	Control	11.03 ± 1.07	437.0 ± 45.0
		Warm	10.06 ± 1.31	465.4 ± 54.8
2015		Control	10.48 ± 0.48	481.4 ± 75.1^∗^
		Warm	10.59 ± 0.76	337.9 ± 32.2
2014	*Machilus breviflora*	Control	11.02 ± 0.89	386.4 ± 80.6
		Warm	10.56 ± 0.76	304.9 ± 118.9
2015		Control	9.93 ± 0.45	380.2 ± 5.66
		Warm	8.42 ± 0.89	409.2 ± 33.4
2014	*Ardisia lindleyana*	Control	17.99 ± 1.00	244.0 ± 47.2^∗∗^
		Warm	16.78 ± 1.97	200.0 ± 49.3
2015		Control	16.52 ± 1.24	311.0 ± 54.4
		Warm	15.43 ± 1.50	242.0 ± 58.1


*^∗^*p* < 0.05; ^∗∗^*p* < 0.01.*

## Discussion

Consistent with our first hypothesis, we observed significant interspecific variation in growth between six species in response to warming, with *S. superba* and *P. massoniana* exhibiting greater increments in growth. *P. massoniana*, which is a native gymnosperm species, has greater competitive ability and growth rates than other coexisting species at the early successive stage (Tang et al., 2011). It has been suggested that warming can stimulate growth of herbaceous monocots, woody gymnosperms and eucalyptus species (McCulloh et al., 2016; Lemoine et al., 2017; Liu et al., 2017; Sharwood et al., 2017). For example, Hou et al. (2011) found that short-term warming stimulated tree growth in height but had no effect on ring width in *Abies faxoniana* seedlings. In *Juniperus thurifera* seedlings, increasing warming temperatures significantly enhanced radial growth rate (Gimeno et al., 2012). Similarly, we observed significant enhancement in height, basal diameter and biomass of *S. superba* and *P. massoniana* in response to warming (**Figure 2**). The enhancement in biomass production of these two species was substantial, being close to 107 and 206% in 2014, respectively. This result was in contradiction to results in previous studies that tropical and subtropical tree species may be near a high temperature threshold (Cunningham and Read, 2002; Clark et al., 2010; Way and Oren, 2010; Bowman et al., 2014), which may result from different plant adaptabilities and local soil water conditions under a warmer environment (Crous et al., 2013). Furthermore, our results showed that the biomass allocation pattern of *P. massoniana* was significantly different between the warm and control sites (**Figure 5**). Roots of *P. massoniana* received greater biomass under warming, whereas there was less biomass allocation to stems and needles. It has been discovered that warming increases the allocation of dry mass to stems, leaves at the expense of roots (Wang et al., 2012; Dawes et al., 2015). In the present study, the enhanced growth performance in response to warming indicated that temperature dominate plant growth, regardless of limited water content. Higher R/S of *P. massoniana* under warming also indicated that in a changing environment the species has certain plasticity in biomass allocation.

In this study, significant increases in SLA of *C. hystrix*, *M. breviflora*, *S. rehderianum*, and *A. lindleyana* were observed under warming (**Figure 4B**), which means that there is a larger amount of leaf area displayed per unit mass in these four species (Poorter et al., 2009). Higher SLA is associated with efficient light capture and could have led to larger assimilation gains (Wang et al., 2012). Significant differences in LAR of *S. rehderianum* and *A. lindleyana* found between warm and control sites indicated that the same amount of whole plant biomass supported a larger leaf area in the warm site (**Figure 4C**). Consistent with our previous studies, from 2012 to 2014, the mean average photosynthetic rates under saturating light for *S. superba*, *M. breviflora*, *P. massoniana*, and *A. lindleyana* in the warm site were 7, 19, 20, and 29% higher under warming (Li et al., 2016a). The warming-induced changes in plant biomass production could have directly resulted from enhanced plant photosynthesis due to higher temperature.

In our study, the leaf starch and soluble sugar content decreased significantly under warming (**Figure 7**). Similar results have been previously reported in other tree species (Deslauriers et al., 2014; Jamieson et al., 2015). The decrease in concentration of carbohydrates may due to the higher foliar respiration rate under warming, which will result in increased consumption of assimilates, such as starch and sugars (Dietze et al., 2014). Soluble sugar and starch accumulation in leaves can have direct effects on photosynthesis through physiological mechanisms (Pritchard et al., 1997), biochemical feedbacks (Rasse and Tocquin, 2006) and gene-expression control (Moore et al., 1999). Furthermore, more carbon may be available to allocate to growth under warming. A previous study suggested that leaf respiration acclimated more strongly than photosynthesis under warming, increasing carbon assimilation but moderating carbon losses (Way and Oren, 2010). Therefore, our results suggest that the response of photosynthesis and carbohydrates allocation contributed directly to the divergent response of tree growth under warming.

Furthermore, the present results showed that warming decreased stomatal density in *S. superba*, *C. hystrix*, and *A. lindleyana*, and had significant effect on stomatal length (**Tables 1**, **2**). Many previous studies have also found warming affected stomatal density and length due to the changes in epidermal cell density (Zheng et al., 2013; Carins Murphy et al., 2016). Lower stomatal density might be induced by elevated temperature, limited water supply and slightly higher vapor pressure deficit (VPD) in the warm site. Our previous studies have reported that the VPD in the warm site was significantly lower than in the control site (Li et al., 2016a). These results suggested that the leaf maximum stomatal conductance and transpiration rate may be constrained by the negative relationship between stomata density and elevated temperature (Franks et al., 2009). It has been previously reported that manipulation of stomatal density could change both instantaneous and long-term water use efficiency (WUE) without altering the photosynthetic capacity (Franks et al., 2015). In our study, significant decrease of leaf δ^13^*C* in *S. rehderianum* and *A. lindleyana* were observed under warming (**Figure 6**), which indicates that warmingdecreased WUE in these two species (Dawson et al., 2002). However, *C. hystrix* under warming showed significantly higher leaf δ^13^*C*-value. In our previous study, we found that warming stimulated photosynthesis in *S. rehderianum* and *A. lindleyana* and decreased it in *C. hystrix* (Li et al., 2016a). Higher WUE in *C. hystrix* under warming has probably been caused by a lowed stomatal conductance and hence a reduced water loss (Li et al., 2016a), together with a reduction in photosynthesis. The decreases of WUE in *S. rehderianum* and *A. lindleyana* under warming may have caused by greater water losses due to increased VPD (León-Sánchez et al., 2016; Li et al., 2016a).

## Conclusion

We found an increase in total biomass production, a shift in the allocation pattern and changes in the physiological in seedlings grown between the warm and the control site for 3 years. The pattern of response was not uniform between species: our data suggest that *S. superba* and *P. massoniana* growth has increased to a greater extent than other species. Therefore, we can conclude that, climate warming may have pronounced effects on trees’ morphology and physiology to support growth under limited soil water condition, inter-specific competition in the subtropical mixed forest. Warmer environment may increase the competitive advantage of *S. superba* and *P. massoniana.* Continued warming may thus lead to changes in the competitive balance and, ultimately, the composition of these mixed forests.

## Author Contributions

JL and GZ conceived and designed the experiments. YL performed the experiments and wrote the main manuscript. All authors contributed to writing and editing the manuscript.

## Conflict of Interest Statement

The authors declare that the research was conducted in the absence of any commercial or financial relationships that could be construed as a potential conflict of interest.
