# Supplementary material for: Different Growth and Physiological Responses of Six Subtropical Tree Species to Warming
Source: Front Plant Sci. 2017 Aug 29;8:1511. doi: 10.3389/fpls.2017.01511 (PMC5583599; doi:10.3389/fpls.2017.01511)
Supplement: Supplementary file 1 [file Table_1.DOC]

**Table S1** Values of regression coefficients (*a* and *b*) in $B=a\text{(}D^{2}H\text{)}+\text{b}$ and *r*^2^, where *B* (g dry mass m^2^) is the biomass of leaf, stem and root of trees. *D* (cm) and *H* (cm) are basal diameter and height of trees, respectively. *r* is correlation coefficient.

| **Species** | **Tissues** | **Equation** | **r^2^** |
| --- | --- | --- | --- |
| *Schima superba* | Root | B= 0.202×(*D*^2^*H*)+15.37 | 0.98 |
|  | Stem | B= 0.478×(*D*^2^*H*)-20.42 | 0.93 |
|  | Leaf | B= 0.337×(*D*^2^*H* )-10.86 | 0.97 |
| *Syzygium rehderianum* | Root | B= 0.140×(*D*^2^*H*)+1.17 | 0.96 |
|  | Stem | B= 0.419×(*D*^2^*H*)-9.06 | 0.91 |
|  | Leaf | B= 0.155×(*D*^2^*H* )+10.63 | 0.91 |
| *Castanopsis* *hystrix* | Root | B= 0.233×(*D*^2^*H*)-3.23 | 0.88 |
|  | Stem | B= 0.404×(*D*^2^*H*)-0.27 | 0.99 |
|  | Leaf | B= 0.215×(*D*^2^*H*)+15.21 | 0.91 |
| *Machilus breviflora* | Root | B= 0.083×(*D*^2^*H*)+5.67 | 0.95 |
|  | Stem | B= 0.368×(*D*^2^*H*)+2.69 | 0.99 |
|  | Leaf | B= 0.183×(*D*^2^*H*)+7.17 | 0.98 |
| *Pinus massoniana* | Root | B= 0.039×(*D*^2^*H*)+5.44 | 0.99 |
|  | Stem | B= 0.119×(*D*^2^*H*)+10.78 | 0.81 |
|  | Leaf | B= 0.072×(*D*^2^*H*)+27.13 | 0.99 |
|  | Root | B= 1.881×(*D*^2^*H*)-4.26 | 0.89 |
| *Ardisia lindleyana* | Stem | B= 0.163×(*D*^2^*H*)+1.36 | 0.92 |
|  | Leaf | B= 1.186×(*D*^2^*H*)-1.44 | 0.89 |
